# Evaluation of executive dysfunction in clinical practice- pragmatic challenges and solutions

**DOI:** 10.1192/j.eurpsy.2021.1359

**Published:** 2021-08-13

**Authors:** D. Vasile, O. Vasiliu

**Affiliations:** Psychiatry, University Emergency Central Military Hospital Dr. Carol Davila, Bucharest, Romania

## Abstract

**Introduction:**

Executive dysfunction (ED) is an important clinical dimension that need to be evaluated in neurologic and psychiatric disorders, due to this factor’s impact over patients functionality, quality of life, and overall prognosis. Working memory, inhibition, set shifting, and fluency are the most widely accepted dimensions of ED. The evaluation of these aspects in clinical practive should be more frequently implemented, because they may offer an image about the patients ability to cope with new situations, stressful events, social challenges being included.

**Objectives:**

To determine the most appropriate methods of ED evaluation in psychiatric practice.

**Methods:**

A literature review was conducted through main electronic databases, and papers published between January 2000 and August 2020 were included in the analysis.

**Results:**

Wisconsin Card Sorting Test (WCST), Halstead-Reitan Neuropsychological Battery, Cambridge Neuropsychological Automated Battery (CANTAB), Wechsler Adult Intelligence Scale (WAIS), and Controlled Oral Word Association Test (COWAT) have been the most frequently cited instruments for ED evaluation. The neuropsychiatric disorders in which ED was recommended in clinical trials were schizophrenia and other psychotic disorders, neurocognitive disorders, bipolar disorders, and major depressive disorders. An integrated approach, that combines initial evaluation and periodic monitoring of symptoms severity, global functioning, quality of life, and ED severity should be considered standard-of-practice for neuropsychiatric disorders.

**Conclusions:**

ED is an important dimension of clinical evaluation in many psychiatric disorders that is useful because the results may correlate to the patients prognosis, quality of life, and overall functional evolution.

**Conflict of interest:**

No significant relationships.

